# Methotrexate Exposure and Inflammatory–Metabolic Biomarker Networks in Hospitalized Patients with Psoriasis: A Network Analysis Approach

**DOI:** 10.3390/ph19050720

**Published:** 2026-05-01

**Authors:** Laura-Florina Nistor, Ruxandra-Cristina Marin, Laura Maria Endres, Gabriela S. Bungau, Ada Radu, Diana Alina Bei, Delia Mirela Tit

**Affiliations:** 1 Doctoral School of Biomedical Sciences, Faculty of Medicine and Pharmacy, University of Oradea, 410087 Oradea, Romaniagbungau@uoradea.ro (G.S.B.); adaradu@uoradea.ro (A.R.); dtit@uoradea.ro (D.M.T.); 2 Department of Pharmacology, Clinical Pharmacology and Pharmacotherapy, Faculty of Medicine, “Carol Davila” University of Medicine and Pharmacy, 050474 Bucharest, Romania; 3 Department of Psycho-Neurosciences and Recovery, Faculty of Medicine and Pharmacy, University of Oradea, 410073 Oradea, Romania; 4 Department of Pharmacy, Faculty of Medicine and Pharmacy, University of Oradea, 410028 Oradea, Romania; 5 Department of Medical Disciplines and Recovery, Faculty of Medicine and Pharmacy, University of Oradea, 410073 Oradea, Romania; diana.bei@didactic.uoradea.ro

**Keywords:** psoriasis, methotrexate, systemic inflammation, insulin resistance, cardiometabolic risk, neutrophil-to-lymphocyte ratio, systemic immune-inflammation index, triglyceride–glucose index, network analysis, EBICglasso

## Abstract

**Background:** Psoriasis is a chronic immune-mediated inflammatory disorder strongly associated with cardiometabolic comorbidities. Although methotrexate (MTX) is widely used for moderate-to-severe disease, its influence on the relationships between inflammatory and metabolic biomarkers remains insufficiently characterized. **Methods:** This retrospective observational study included 132 hospitalized adult patients with psoriasis, stratified into untreated (n = 101) and MTX-treated (n = 31) groups. Inflammatory markers, C-reactive protein (CRP), erythrocyte sedimentation rate (ESR), neutrophil-to-lymphocyte ratio (NLR), and systemic immune-inflammation index (SII), and metabolic indices, triglyceride–glucose index (TyG), metabolic score for insulin resistance (METS-IR), and atherogenic index of plasma (AIP), were analyzed. Group comparisons were performed using Mann–Whitney U and χ^2^ tests. Spearman correlation matrices and regularized partial correlation networks (EBICglasso, γ = 0.5) were constructed separately for each group to explore inflammatory–metabolic connectivity. **Results:** MTX-treated patients exhibited lower NLR (*p* = 0.035) and fasting glucose levels (*p* = 0.004), while CRP, ESR, and composite metabolic indices did not differ significantly. In untreated patients, correlation analysis showed multiple significant cross-domain associations between inflammatory and metabolic markers. In contrast, fewer such associations reached statistical significance in the MTX-treated group. Network analysis indicated a less densely connected structure in the MTX group (9 vs. 12 non-zero edges); however, formal network comparison did not identify statistically significant differences between groups. **Conclusions**: Although fewer statistically significant cross-domain correlations were observed in MTX-treated patients, no statistically significant differences in network structure were detected between groups. These findings are exploratory and hypothesis-generating, not indicative of methotrexate-related modification of network structure, and are limited by the small size of the MTX-treated subgroup.

## 1. Introduction

Psoriasis is a chronic immune-mediated inflammatory disease that primarily affects the skin but is increasingly recognized as a systemic disorder with multisystem implications. The disease affects approximately 2–3% of the global population corresponding to more than 125 million people worldwide, making it one of the most common chronic dermatologic conditions. Prevalence varies geographically, ranging from less than 1% in some regions to over 8–11% in Northern European countries, reflecting genetic, environmental, and demographic differences [1,2].

Beyond the characteristic erythematous and scaly plaques, psoriasis imposes a considerable physical and psychosocial burden and is associated with reduced quality of life and increased healthcare utilization. It is now widely considered a systemic inflammatory disease that predisposes patients to multiple comorbidities, particularly cardiometabolic disorders that contribute substantially to long-term morbidity and mortality [3]. Thus, psoriasis is characterized by complex interactions between immune, metabolic, and vascular pathways, which are increasingly recognized as key contributors to the broad spectrum of disease-associated comorbidities [4].

The pathogenesis of psoriasis involves complex interactions between genetic susceptibility, environmental triggers, and dysregulated immune responses. Central to disease development is the activation of innate and adaptive immune pathways, particularly the interleukin-23/interleukin-17 (IL-23/IL-17) axis and tumor necrosis factor-α (TNF-α) signaling, which are now recognized as major drivers of psoriatic inflammation [5,6]. These cytokine networks promote keratinocyte hyperproliferation, endothelial activation, and recruitment of inflammatory cells to the skin, leading to the formation of characteristic psoriatic plaques [7]. The inflammatory cascade involves interactions between dendritic cells, T helper 17 (Th17) cells, and keratinocytes, resulting in sustained production of pro-inflammatory mediators such as IL-17, IL-22, and TNF-α that maintain chronic skin inflammation [6]. These immune pathways exert systemic effects beyond the skin, contributing to low-grade systemic inflammation and supporting the concept that psoriasis represents a systemic inflammatory disorder rather than a purely dermatologic disease [8].

Recent evidence indicates that chronic inflammation in psoriasis contributes to metabolic dysregulation and increased cardiovascular risk. Persistent activity of pro-inflammatory cytokines such as tumor necrosis factor-α (TNF-α), interleukin-17 (IL-17), and interleukin-23 (IL-23) may interfere with insulin signaling pathways, promote adipose tissue inflammation, and induce endothelial dysfunction, thereby facilitating the development of insulin resistance, dyslipidemia, and atherosclerosis [9].

Epidemiological studies have consistently demonstrated a higher prevalence of metabolic syndrome, obesity, hypertension, and type 2 diabetes mellitus among individuals with psoriasis compared with the general population. These cardiometabolic conditions share common inflammatory and metabolic pathways with psoriasis, supporting the concept of a bidirectional relationship between immune activation and metabolic dysfunction [10,11,12]. Furthermore, psoriasis itself has been identified as an independent cardiovascular risk factor associated with ischemic heart disease, stroke, and other vascular complications [4].

Systemic inflammatory activity in psoriasis can be evaluated using circulating biomarkers. Acute-phase reactants such as C-reactive protein (CRP) and erythrocyte sedimentation rate (ESR) are commonly used indicators of systemic inflammation and have been linked to both disease severity and cardiovascular risk [13,14]. In addition, hematologic indices derived from routine blood counts have gained increasing attention as accessible markers of immune balance. Among these, the neutrophil-to-lymphocyte ratio (NLR) reflects the relative predominance of innate inflammatory responses over adaptive immunity, while the systemic immune-inflammation index (SII), calculated using neutrophil, lymphocyte, and platelet counts, integrates multiple components of the inflammatory response and may provide a broader representation of immune activation [15].

In parallel, metabolic disturbances associated with psoriasis can be assessed using surrogate markers of insulin resistance and atherogenic risk derived from routine biochemical parameters. Indices such as the triglyceride–glucose (TyG) index, metabolic score for insulin resistance (METS-IR), and atherogenic index of plasma (AIP) integrate lipid and glucose metabolism to estimate metabolic dysfunction and cardiovascular risk. These markers have been increasingly used in clinical research as practical tools for evaluating cardiometabolic risk in populations with chronic inflammatory diseases [8].

Although inflammatory and metabolic biomarkers have been widely investigated in psoriasis, most studies have traditionally examined these parameters independently. However, inflammation and metabolism represent closely interconnected biological processes that influence each other through complex immunometabolic pathways. Specifically, adipose tissue functions as an active endocrine and immune organ capable of secreting adipokines and cytokine-like mediators, including leptin, resistin, adiponectin, and inflammatory cytokines such as IL-6 and TNF-α. These mediators can modulate both systemic metabolic homeostasis and immune responses, thereby contributing to chronic low-grade inflammation observed in psoriasis [16,17]. Recent experimental evidence indicates that psoriasis-related cytokines, especially IL-17 and TNF-α, can alter adipose tissue gene expression and secretory profiles, promoting a pro-inflammatory adipose microenvironment that further amplifies systemic inflammation and metabolic dysfunction [17].

In this context, adipocytokine imbalance and adipose tissue inflammation may establish a bidirectional pathogenic loop in which metabolic alterations aggravate cutaneous inflammation while systemic inflammatory signals promote metabolic disturbances. Consequently, inflammatory and metabolic pathways in psoriasis are increasingly understood as components of an integrated biological network in which dysregulation within one domain can influence the other [18,19]. This growing recognition of psoriasis as an immunometabolic disease highlights the need for analytical approaches capable of capturing interactions between multiple biological domains simultaneously. Systems-level approaches, including network-based analyses, have recently emerged as valuable tools for exploring complex biological interactions and identifying key nodes that may contribute to disease progression or therapeutic response [20,21].

Systemic therapy has the potential to influence not only the magnitude of inflammatory activity but also the broader organization of inflammatory–metabolic interactions. Methotrexate (MTX) remains one of the most widely used systemic therapies for moderate-to-severe psoriasis due to its established efficacy, relatively low cost, and long clinical experience [22]. MTX exerts anti-inflammatory effects primarily through inhibition of folate-dependent metabolic pathways, increased extracellular adenosine signaling, and modulation of T-cell activation and cytokine production, including suppression of TNF-α, IL-6, and other pro-inflammatory mediators involved in psoriatic inflammation. Through these mechanisms, MTX reduces immune activation and keratinocyte proliferation while contributing to the attenuation of systemic inflammatory responses [23]. Moreover, methotrexate (MTX) therapy may reduce cardiovascular risk in inflammatory diseases by lowering systemic inflammation and improving endothelial function. Evidence suggests that MTX exerts anti-inflammatory and anti-atherogenic effects that may slow the progression of atherosclerosis [24].

Observational studies in psoriasis and other chronic inflammatory conditions have reported associations between methotrexate exposure and a reduced incidence of cardiovascular events, supporting the hypothesis that effective anti-inflammatory therapy may partially mitigate inflammation-driven cardiometabolic risk [25,26]. Despite these observations, the extent to which MTX therapy modifies the structural relationships between inflammatory and metabolic processes remains insufficiently explored. Most clinical studies evaluating systemic therapies in psoriasis have focused primarily on changes in individual inflammatory or metabolic biomarkers, while the broader interactions between these biological domains remain less clearly characterized [8].

Traditional statistical analyses in clinical research typically focus on differences in individual biomarker levels between treatment groups. While such approaches provide important information, they may not fully capture the complexity of biological systems in which multiple variables interact simultaneously. Network analysis offers a complementary methodological framework that models conditional dependencies between variables and enables evaluation of how relationships among biomarkers may reorganize under different biological conditions or therapeutic interventions [27,28].

By examining patterns of interconnections among inflammatory and metabolic biomarkers rather than isolated marker levels, network modeling may provide additional insight into how systemic therapies influence the broader biological architecture of disease-related processes. Such approaches are particularly relevant in chronic inflammatory disorders, where immune and metabolic pathways are closely interconnected. However, the extent to which systemic therapies modify inflammatory–metabolic interactions in psoriasis remains insufficiently characterized. Therefore, the present study aimed to explore differences in inflammatory and metabolic biomarker profiles in hospitalized patients with psoriasis according to methotrexate exposure, and to examine whether distinct patterns of connectivity between these domains can be identified using correlation and network-based approaches.

## 2. Results

### 2.1. Baseline Characteristics

Baseline demographic and clinical characteristics of untreated patients and those receiving systemic methotrexate are presented in Table 1. No statistically significant differences were observed between groups with regard to sex distribution (*p* = 0.515) or area of residence (urban/rural; *p* = 0.222). Similarly, the prevalence of major cardiometabolic comorbidities—including obesity (*p* = 0.774), diabetes mellitus (*p* = 0.943), dyslipidemia (*p* = 0.656), other cardiovascular disease (*p* = 0.497), chronic kidney disease (*p* = 0.543), and metabolic syndrome (*p* = 0.544)—did not differ significantly between groups. Hypertension was more frequent among MTX-treated patients (74.2% vs. 55.4%); however, this difference did not reach statistical significance (*p* = 0.063).

Continuous variables were also broadly similar between groups. Age (*p* = 0.386), disease onset (*p* = 0.252), and length of hospital stay (*p* = 0.581) did not differ significantly. In contrast, the number of prior hospitalizations was significantly higher in the MTX group (*p* = 0.006).

Overall, the two groups showed broadly comparable baseline cardiometabolic profiles, although MTX-treated patients had a higher frequency of previous hospitalizations.

Among MTX-treated patients (n = 31), weekly doses ranged from 7.5 to 22.5 mg. Most patients received 15 mg/week (45.2%) or 20 mg/week (32.3%), while lower doses (≤12.5 mg) and 22.5 mg were infrequent. Overall, 77% of patients were treated within the 15–20 mg/week range.

### 2.2. Inflammatory and Metabolic Biomarkers

The inflammatory and metabolic biomarker levels (according to methotrexate exposure) are summarized in Table 2. Overall, most inflammatory markers were comparable between untreated patients and those receiving MTX therapy. CRP (*p* = 0.830), ESR (*p* = 0.234), and the systemic immune-inflammation index (SII) (*p* = 0.569) did not differ significantly between groups.

In contrast, the neutrophil-to-lymphocyte ratio (NLR) was significantly lower in MTX-treated patients compared with untreated individuals (*p* = 0.035; rank-biserial correlation = 0.252), indicating a difference between groups. Absolute leukocyte parameters showed no meaningful differences between groups. Total leukocyte counts, neutrophil counts, lymphocyte counts, and HDL cholesterol levels were similar in both groups (all *p* > 0.05). Platelet counts tended to be higher in MTX-treated patients; however, this difference did not reach statistical significance (*p* = 0.072).

Regarding metabolic parameters, fasting glucose levels were significantly lower in MTX-treated patients (median 95 [84–108.5] mg/dL) compared with untreated individuals (median 111 [94–131] mg/dL; *p* = 0.004; rank-biserial correlation = 0.347). However, composite metabolic indices reflecting insulin resistance and atherogenic risk, including TyG (*p* = 0.629), METS-IR (*p* = 0.691), and AIP (*p* = 0.947), were comparable between groups.

Overall, MTX-treated patients had lower NLR and fasting glucose levels, while broader inflammatory markers and composite metabolic indices remained largely similar between the two groups.

Within the MTX-treated subgroup (n = 31), weekly doses ranged from 7.5 to 22.5 mg, with most patients receiving doses between 15 and 20 mg/week (77%). Spearman correlation analysis did not demonstrate significant associations between MTX dose and inflammatory markers (NLR, SII, ESR, CRP) or metabolic indices (TyG, AIP, METS-IR) (all *p* > 0.05). Given the relatively narrow dose distribution and limited sample size, potential dose–response relationships could not be reliably assessed.

### 2.3. Correlation Patterns According to Methotrexate Exposure

**Figure 1:**
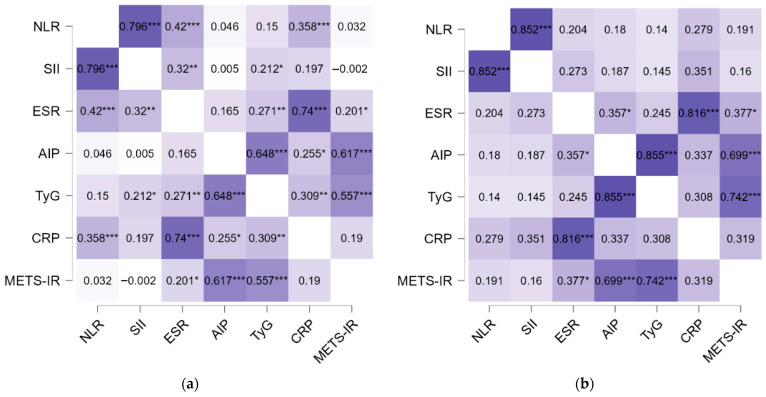
Spearman correlation heatmaps of inflammatory and metabolic biomarkers in no therapy (**a**) and methotrexate-treated (**b**) patients. Color intensity reflects correlation strength (ρ), with darker shades indicating stronger correlations. Statistical significance is indicated as * *p* < 0.05, ** *p* < 0.01, *** *p* < 0.001. NLR, neutrophil-to-lymphocyte ratio; SII, systemic immune-inflammation index; ESR, erythrocyte sedimentation rate; CRP, C-reactive protein; TyG, triglyceride–glucose index; AIP, atherogenic index of plasma; METS-IR, metabolic score for insulin resistance.

Spearman correlation matrices were constructed separately for untreated patients (n = 101) and those receiving systemic methotrexate (n = 31) to explore inflammatory–metabolic interdependencies (Table 3 and Figure 1).

In the untreated patients, strong intra-inflammatory coupling was observed. NLR showed a very strong positive correlation with SII (ρ = 0.796, *p* < 0.001), while CRP correlated strongly with ESR (ρ = 0.740, *p* < 0.001), confirming consistent associations among inflammatory markers.

Metabolic indices were also closely interconnected. TyG demonstrated strong correlations with both AIP (ρ = 0.648, *p* < 0.001) and METS-IR (ρ = 0.557, *p* < 0.001), reflecting coherent clustering of metabolic risk indicators.

Several cross-domain associations linking inflammation and metabolism were also observed. CRP correlated significantly with TyG (ρ = 0.309, *p* = 0.002) and AIP (ρ = 0.255, *p* = 0.011), while ESR showed moderate correlations with TyG (ρ = 0.271, *p* = 0.006) and METS-IR (ρ = 0.201, *p* = 0.044). These findings are consistent with an integrated inflammatory–metabolic profile in untreated patients.

In MTX-treated patients, strong intra-domain correlations remained evident. NLR maintained a very strong association with SII (ρ = 0.852, *p* < 0.001), and CRP continued to correlate robustly with ESR (ρ = 0.816, *p* < 0.001).

Similarly, metabolic indices remained tightly interconnected, particularly TyG with AIP (ρ = 0.855, *p* < 0.001) and METS-IR (ρ = 0.742, *p* < 0.001), as well as AIP with METS-IR (ρ = 0.699, *p* < 0.001).

However, several cross-domain correlations observed in untreated patients did not retain statistical significance in the MTX subgroup. For instance, the correlations between CRP and TyG (ρ = 0.308, *p* = 0.098) and between CRP and AIP (ρ = 0.337, *p* = 0.069) were not statistically significant. NLR also showed no significant correlations with metabolic indices in this subgroup.

ESR maintained modest correlations with AIP (ρ = 0.357, *p* = 0.049) and METS-IR (ρ = 0.377, *p* = 0.037), indicating partial persistence of inflammatory–metabolic associations.

Overall, untreated patients exhibited broader statistically significant cross-domain correlations linking inflammatory and metabolic markers, whereas these associations were less consistently detected in the MTX-treated subgroup. However, the smaller size of the MTX-treated subgroup may have reduced statistical power to detect correlations, and therefore the absence of statistical significance should be interpreted with caution.

### 2.4. Network Analysis According to Methotrexate Exposure

To examine conditional interdependencies among inflammatory and metabolic biomarkers, regularized partial correlation networks were estimated separately for untreated patients and those receiving systemic methotrexate using the EBICglasso method (tuning parameter γ = 0.5). Both networks consisted of six nodes representing inflammatory and metabolic biomarkers (NLR, SII, ESR, CRP, TyG, and METS-IR).

The network estimated in untreated patients retained 12 of the 15 possible edges, resulting in a relatively dense connectivity structure (sparsity = 0.20). In contrast, the MTX-treated network retained 9 of the 15 possible edges (sparsity = 0.40), corresponding to a sparser connectivity pattern. The MTX network retained fewer edges but showed a slightly higher mean edge weight (Table 4).

**Figure 2:**
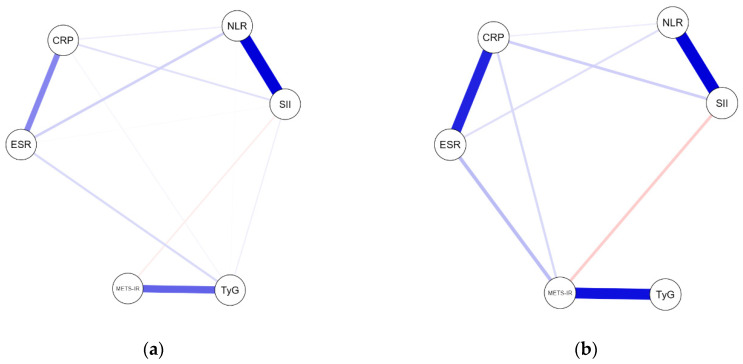
EBICglasso regularized partial correlation networks of inflammatory and metabolic biomarkers in untreated patients (**a**) and methotrexate-treated patients (**b**); nodes represent biomarkers and edges represent regularized partial correlations between variables. Edge thickness corresponds to the magnitude of the association. Only non-zero edges retained after regularization are displayed; NLR, neutrophil-to-lymphocyte ratio; SII, systemic immune-inflammation index; ESR, erythrocyte sedimentation rate; CRP, C-reactive protein; TyG, triglyceride–glucose index; METS-IR, metabolic score for insulin resistance.

In untreated patients (Figure 2a), inflammatory and metabolic markers formed a highly interconnected structure. The strongest partial correlations were observed between CRP and TyG (0.870), ESR and METS-IR (0.535), and NLR and SII (0.418). Several cross-domain connections linking inflammatory and metabolic markers were present, particularly the strong CRP–TyG edge, representing one of the strongest cross-domain edges in the network.

In the MTX-treated network (Figure 2b), core inflammatory coupling between NLR and SII remained evident with a higher edge weight (0.580). The ESR–METS-IR association also persisted (0.621).

Fewer cross-domain edges were retained following regularization, resulting in a network with fewer retained connections. Given the relatively small size of the MTX-treated subgroup, differences in edge retention may partly reflect sample size-related variability and reduced estimation stability rather than true underlying differences. The CRP–TyG association remained one of the dominant cross-domain edges in both networks.

Centrality measures based on node strength indicated that inflammatory indices were highly central within the untreated network. Particularly, NLR and SII exhibited the highest strength values, highlighting their role as central nodes within the inflammatory cluster. In the MTX-treated network, METS-IR and SII showed relatively higher strength values. Betweenness and closeness centrality measures were not interpreted due to their known instability in small samples, and differences in centrality between networks should be considered descriptive.

Edge accuracy and centrality stability were evaluated using nonparametric bootstrap procedures (1000 resamples). Bootstrap analyses indicated that the strongest edges (CRP–TyG, ESR–METS-IR, and NLR–SII) demonstrated acceptable stability, whereas weaker connections showed greater variability. Case-dropping bootstrap analysis suggested that edge stability was acceptable in the untreated group but reduced in the MTX-treated subgroup. Strength centrality demonstrated moderate stability in the untreated network, whereas centrality measures in the MTX-treated network were unstable, limiting their interpretability.

A formal comparison between networks using the network comparison test (NCT) with 1000 permutations did not reveal statistically significant differences in network structure (M = 0.19, *p* = 0.959) or global strength (S = 0.097, *p* = 0.910) between groups.

Overall, the estimated networks showed fewer retained edges in the MTX-treated subgroup compared with untreated patients, while several intra-domain associations remained evident. However, formal network comparison did not identify statistically significant differences in global structure or strength between groups. Therefore, these patterns should be interpreted as descriptive and exploratory representations of the observed data rather than evidence of differences in network structure attributable to methotrexate exposure. Given the cross-sectional design and the relatively small MTX-treated subgroup (n = 31), these findings should be interpreted with caution, as limited sample size may affect edge stability and the ability to detect true differences between networks, and do not support causal or inferential conclusions.

## 3. Discussion

The present study examined inflammatory–metabolic interactions in hospitalized patients with psoriasis according to methotrexate (MTX) exposure using complementary analytical approaches, including correlation analysis and regularized partial correlation network modeling. Baseline demographic and cardiometabolic characteristics were comparable between groups, and the findings provide a descriptive comparison of biomarker interaction patterns. Due to relatively small size of the MTX-treated subgroup, results should be interpreted with caution, as limited statistical power may affect the stability of correlation estimates and network models. In the absence of statistically significant differences in formal network comparison and considering the cross-sectional design, these findings are descriptive and exploratory. The observed differences in cross-domain correlations and retained edges should not be interpreted as evidence of methotrexate-related changes in inflammatory–metabolic connectivity.

Psoriasis is increasingly recognized as a systemic inflammatory disease associated with multiple cardiometabolic comorbidities, including obesity, hypertension, dyslipidemia, insulin resistance, and metabolic syndrome. These associations are driven by shared inflammatory pathways involving cytokines such as tumor necrosis factor-α, IL-6, and IL-17, which influence both immune activity and metabolic homeostasis. Consequently, psoriasis is now considered an inflammatory–metabolic disorder in which chronic systemic inflammation contributes to metabolic dysfunction and increased cardiovascular risk, with meta-analyses confirming a higher prevalence of metabolic syndrome among affected patients [29].

Studies have further emphasized the systemic inflammatory–metabolic nature of psoriasis. Analyses integrating biochemical and clinical data demonstrate that patients frequently exhibit concurrent elevations of inflammatory biomarkers and metabolic abnormalities, supporting the concept of a shared inflammatory–metabolic axis contributing to disease burden and cardiometabolic risk [18].

These observations are in line with the interconnected inflammatory–metabolic network observed in our untreated cohort, where multiple correlations linked inflammatory indices with metabolic markers.

In the present cohort, cardiometabolic comorbidities were similarly distributed between untreated and methotrexate-treated patients, suggesting that the observed differences in biomarker interactions were unlikely to be driven by baseline metabolic disparities. The higher number of previous hospitalizations observed in the MTX group may reflect greater cumulative disease burden or more severe disease requiring systemic therapy, which could also be associated with differences in inflammatory and metabolic profiles independent of current treatment status. This observation aligns with current clinical practice, where MTX remains one of the most widely used first-line systemic agents for moderate-to-severe psoriasis due to its established efficacy, accessibility, and long-standing clinical experience [30].

Differences at the level of individual biomarkers were limited, with only selected parameters showing variation between groups. NLR is increasingly recognized as a surrogate marker of systemic inflammatory activity and immune imbalance in psoriasis and other chronic inflammatory conditions. Elevated NLR reflects increased neutrophil-mediated innate immune activation accompanied by relative lymphocyte suppression, processes that contribute to systemic inflammatory burden. Clinical studies have demonstrated that NLR is significantly increased in patients with psoriasis and correlates with disease activity and systemic inflammatory burden [31].

Growing evidence indicates that blood-count-derived inflammatory indices represent practical and accessible tools for monitoring systemic inflammation. Indices such as NLR, platelet-to-lymphocyte ratio, and systemic immune-inflammation index have been shown to change during systemic therapy and may reflect treatment-induced immunomodulation [32].

The lower NLR values observed in MTX-treated patients may reflect the immunomodulatory effects of systemic therapy. Methotrexate exerts known immunomodulatory effects, including modulation of cytokine production and immune cell function [33]. From a pharmacological perspective, methotrexate exerts complex immunomodulatory effects that may influence both inflammatory and metabolic pathways; however, such effects may not be fully captured in cross-sectional biomarker analyses.

Despite the reduction in NLR, classical acute-phase reactants such as CRP and ESR did not differ significantly between the groups. This discrepancy may reflect the complex kinetics and limited specificity of these markers in hospitalized populations, where multiple factors (infections, comorbid conditions, or physiological stress) may influence systemic inflammatory protein levels. In contrast, cellular inflammatory indices derived from leukocyte counts may capture immune system dynamics more sensitively in chronic inflammatory diseases such as psoriasis. Recent studies suggest that hematological indices such as NLR reflect systemic inflammatory activity and correlate with disease severity and inflammatory burden in psoriasis [34].

Additionally, residual systemic inflammation may persist even in patients with clinically controlled psoriasis. Cross-sectional analyses have shown that markers such as NLR and CRP may remain elevated compared with healthy controls despite successful therapy [35].

Another important finding of the present study was the significantly lower fasting glucose level observed in methotrexate-treated patients compared with untreated individuals. Chronic systemic inflammation contributes to insulin resistance through cytokine-mediated disruption of insulin signaling pathways in adipose tissue and skeletal muscle. Inflammatory mediators such as TNF-α, IL-6, and IL-17 have been implicated in metabolic dysregulation and impaired glucose metabolism in immune-mediated inflammatory diseases including psoriasis and psoriatic arthritis [36].

Modulation of inflammatory pathways has been associated with metabolic regulation. Evidence indicates that modulation of immune pathways can influence metabolic homeostasis through interactions between inflammatory cytokines, adipokines, and metabolic signaling networks [37].

However, composite metabolic indices, such as TyG, METS-IR, and AIP, did not differ significantly between groups in our cohort. These indices integrate multiple metabolic parameters and may reflect longer-term metabolic disturbances that are less sensitive to modest changes in fasting glucose observed in cross-sectional analyses. Nevertheless, their inclusion remains clinically relevant, as markers such as the TyG index have been associated with cardiovascular risk and metabolic dysfunction in psoriasis [38].

Importantly, these network-level differences occurred in the context of relatively modest changes in individual biomarkers, limited primarily to NLR and fasting glucose. This apparent discrepancy may indicate differences in relationships between variables.

Large observational cohorts have demonstrated strong links between metabolic dysfunction and systemic inflammatory activity in psoriasis, highlighting the bidirectional relationship between immune activation and metabolic dysregulation [39].

Several clinical factors may have influenced the observed inflammatory–metabolic relationships. Disease severity, for example, is known to affect both systemic inflammatory burden and metabolic dysregulation [18]. Patients with more severe psoriasis may exhibit stronger inflammatory–metabolic coupling, which could influence both correlation patterns and network connectivity.

Similarly, the duration of methotrexate therapy and cumulative exposure may influence immune and metabolic pathways over time. Pharmacokinetic and pharmacodynamic studies indicate that cumulative exposure, including intracellular methotrexate polyglutamate accumulation, may influence therapeutic effects and biomarker profiles [40]. In addition, concomitant medications, including systemic or topical therapies and treatments for cardiometabolic comorbidities, may independently influence inflammatory and metabolic markers, thereby affecting both individual biomarker levels and their interrelationships [41].

As these variables were not fully captured in the present study, the observed patterns may partly reflect underlying clinical heterogeneity rather than methotrexate exposure alone. Therefore, the findings should be interpreted with caution, and future studies incorporating detailed treatment history and disease severity measures are needed to better disentangle these effects.

In untreated patients, strong correlations between inflammatory and metabolic markers were observed. Strong intra-domain correlations between inflammatory markers (e.g., NLR and SII, CRP and ESR) and metabolic indices (TyG, AIP, METS-IR) indicated consistent within-domain associations. In addition, statistically significant cross-domain correlations were observed, consistent with previously reported associations between systemic inflammation and metabolic dysfunction in psoriasis [42].

In contrast, fewer statistically significant cross-domain associations were observed in MTX-treated patients. While intra-domain relationships remained evident within inflammatory and metabolic clusters, the reduced number of statistically significant correlations in the MTX-treated subgroup may partly reflect the relatively small sample size and limited statistical power and should be interpreted with caution. Similar observations have been reported in studies indicating that systemic immunomodulatory therapies can modify inflammatory activity while exerting variable effects on metabolic regulation [43].

Network modeling provided a complementary perspective by highlighting relationships between biomarkers rather than changes in their individual levels. Network analysis using EBICglasso estimates conditional dependencies between variables and allows exploration of potential interaction patterns; however, such models are inherently exploratory and do not establish causal relationships. Although internal relationships within inflammatory and metabolic clusters remained evident, fewer cross-domain edges were retained after regularization. These patterns should be interpreted cautiously, as they were not supported by statistically significant differences in formal network comparison and may be influenced by sample size and model regularization. In particular, the limited size of the MTX-treated subgroup may influence network sparsity due to sample size and regularization effects.

Formal comparison using the network comparison test did not reveal statistically significant differences in global network structure or strength between groups. This finding should be interpreted in the context of limited statistical power, particularly given the relatively small size of the MTX-treated subgroup. Under these conditions, the ability to detect true differences in network structure is reduced, and non-significant results do not imply equivalence between networks. Although regularized approaches such as EBICglasso reduce overfitting by shrinking weaker associations, they do not fully compensate for limited sample size, and network structures remain sensitive to sampling variability. Bootstrap procedures were used to assess edge accuracy and centrality stability; however, stability was reduced in the MTX-treated subgroup, reflecting the limited sample size. Accordingly, the estimated networks should be interpreted as exploratory representations of conditional dependencies rather than definitive models of biological structure. These analyses do not support inference regarding causality, directionality, or treatment effects, nor do they provide statistically confirmed evidence of differences between groups. The findings should therefore be considered hypothesis-generating and require validation in larger studies.

Overall, fewer cross-domain associations were observed in the MTX-treated subgroup, alongside preserved intra-domain relationships. Causal interpretations cannot be established in this cross-sectional analysis. Recent molecular and proteomic studies demonstrate that immune and metabolic pathways may form partially distinct yet interacting modules within inflammatory diseases [44]. Systems biology approaches highlight how biological networks may vary even when individual biomarkers change modestly [45].

No significant associations were identified between MTX dose and inflammatory or metabolic biomarkers within the treated subgroup. This finding should be interpreted cautiously given the relatively narrow dose distribution in the cohort. Low-dose methotrexate within a range of approximately 10–30 mg/week represents the standard systemic dosing strategy used in immune-mediated inflammatory diseases, including psoriasis [40].

Limited variability in weekly dosing may therefore reduce the ability to detect dose–response relationships in cross-sectional analyses. Moreover, pharmacokinetic studies indicate that intracellular MTX polyglutamate accumulation (which reflects cumulative exposure) plays an important role in determining pharmacodynamic effects and therapeutic response [46].

From a clinical perspective, these findings do not have direct implications for clinical decision-making but suggest that network-based approaches may provide complementary insight into the systemic organization of inflammatory and metabolic processes in psoriasis. Any potential clinical relevance remains speculative and requires validation in prospective studies. Chronic systemic inflammation contributes to endothelial dysfunction and accelerated atherosclerosis, which partly explains the increased cardiovascular morbidity observed in this population [26]. Recent epidemiological studies further highlight the role of persistent inflammatory activity in vascular pathology and cerebrovascular risk among patients with psoriasis [47]. These findings suggest that network-based approaches may provide complementary insight into the systemic organization of inflammatory and metabolic processes in psoriasis. In the future, such approaches may contribute to risk stratification or identification of patient subgroups, although this remains to be established.

However, the metabolic effects of systemic therapies appear heterogeneous. Prospective clinical investigations comparing methotrexate with other systemic treatments have reported significant improvements in disease activity but relatively modest or inconsistent changes in metabolic parameters [48]. This is consistent with our findings, where fasting glucose differed between groups while composite metabolic indices remained largely unchanged. At the network level, fewer cross-domain interactions and preserved intra-domain relationships were observed. In the absence of statistically significant differences and given the methodological constraints of the study, these observations should be interpreted as descriptive and hypothesis-generating rather than indicative of confirmed bio-logical differences.

Several limitations should be considered when interpreting these findings. The cross-sectional design does not allow conclusions regarding causality or temporal changes following treatment initiation. In addition, the relatively small number of MTX-treated patients may have limited statistical power and affected the stability of network estimates, particularly for weaker edges. Important clinical factors, such as disease severity, duration and cumulative exposure to methotrexate, as well as concomitant treatments, were not fully captured and may have influenced both biomarker levels and their interrelationships. Centrality stability, particularly in the MTX-treated subgroup, was limited, likely reflecting the relatively small sample size and restricting the interpretability of centrality-based findings. Furthermore, the absence of statistically significant differences in network comparison testing may be attributable to limited statistical power. Differences in correlation patterns and network structure between groups may also partly reflect sample size imbalance rather than true biological divergence. From a methodological perspective, although regularized network models reduce the risk of overfitting, they remain sensitive to parameter selection and may yield unstable estimates in smaller samples, particularly for weaker associations. Residual confounding cannot be excluded, as unmeasured or incompletely captured variables may have influenced both inflammatory and metabolic markers. Therefore, the observed network structures should be interpreted as exploratory representations of potential relationships rather than definitive models of biological interactions. In this context, network analysis should be viewed as a complementary exploratory tool that can highlight potential patterns of association but cannot establish definitive structural or causal relationships in the present sample.

Despite these limitations, the convergence between correlation analysis and network modeling provides a consistent descriptive perspective. Both approaches suggested fewer statistically significant cross-domain correlations and fewer retained cross-domain edges under methotrexate exposure, while preserving strong intra-domain relationships. These findings reflect differences in observed inflammatory–metabolic connectivity patterns between MTX-treated and untreated patients, without implying causal effects of therapy.

## 4. Materials and Methods

### 4.1. Study Design and Population

This retrospective observational study included consecutive adult patients hospitalized with a discharge diagnosis of psoriasis during the predefined study period at a tertiary care center. The study population was identified through review of the institutional electronic medical database. Consecutive adult patients hospitalized with a discharge diagnosis of psoriasis during the predefined study period were identified through the institutional electronic medical database and screened for eligibility based on predefined inclusion and exclusion criteria.

Patients were eligible if they were ≥18 years of age and had complete laboratory data required for the calculation of inflammatory and metabolic indices, including CRP, ESR, complete blood count with differential, fasting glucose, triglycerides, and HDL cholesterol. Patients receiving systemic therapies other than methotrexate, those treated exclusively with topical agents, and individuals with incomplete laboratory data were excluded.

**Figure 3:**
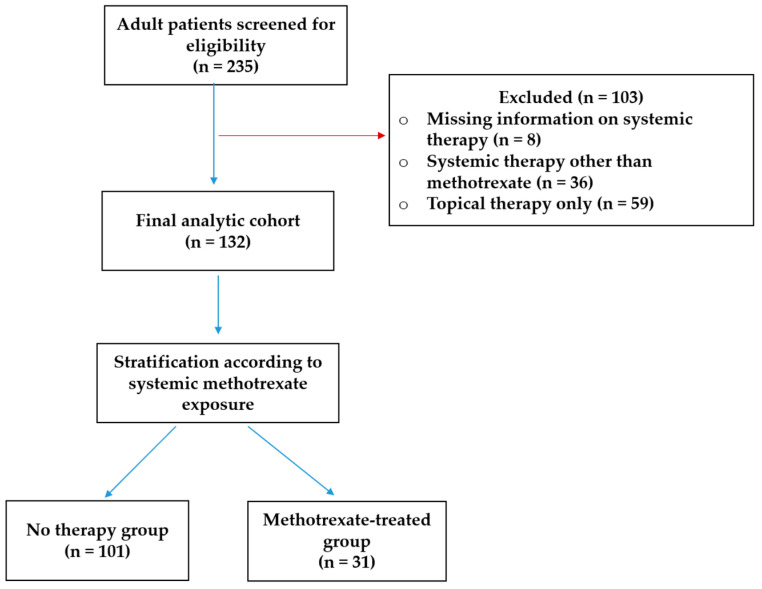
Flow diagram of patients’ selection and cohort stratification in the study population.

After application of these criteria, the final analytic cohort consisted of 132 patients. Participants were stratified according to systemic methotrexate exposure at the time of hospitalization into a no systemic therapy group (n = 101) and a methotrexate-treated group (n = 31). The cohort derivation process is illustrated in Figure 3.

### 4.2. Clinical and Laboratory Variables 

Demographic characteristics, comorbidities, hospitalization data, and treatment information were extracted from electronic medical records. Cardiometabolic comorbidities included hypertension, diabetes mellitus, dyslipidemia, chronic kidney disease, other cardiovascular disease, obesity, and metabolic syndrome. Laboratory variables comprised C-reactive protein (CRP), erythrocyte sedimentation rate (ESR), leukocyte count and differential, platelet count, fasting glucose, triglycerides, and HDL cholesterol. Derived indices were calculated using standard formulas and included the neutrophil-to-lymphocyte ratio (NLR), systemic immune-inflammation index (SII), triglyceride–glucose index (TyG), metabolic score for insulin resistance (METS-IR), and atherogenic index of plasma (AIP). For patients receiving methotrexate, only the weekly dose at the time of admission was consistently available and was therefore included in the analysis. Data regarding treatment duration, cumulative exposure, and erythrocyte methotrexate concentrations were not systematically recorded and could not be analyzed.

Information on concomitant medications and psoriasis severity (e.g., PASI) was not consistently available in the medical records and was therefore not included in the analysis.

### 4.3. Statistical Analysis

Continuous variables were assessed for distributional characteristics and, given non-normal distributions, were compared between groups using the Mann–Whitney U test. Effect sizes for nonparametric comparisons were expressed as rank-biserial correlations. Categorical variables were analyzed using the χ^2^ test. A two-sided *p*-value < 0.05 was considered statistically significant. Given the absence of established methods for formal power calculation in regularized network models and permutation-based network comparison tests, no a priori or post hoc power analysis was performed. Accordingly, statistical findings, particularly those derived from network analysis, were interpreted cautiously in light of the exploratory design and the relatively small MTX-treated subgroup. Regularized network modeling was used as an exploratory systems-level approach to evaluate conditional dependencies among biomarkers; these analyses were not intended to support confirmatory inference but to generate hypotheses regarding interaction patterns.

To evaluate inflammatory–metabolic interdependencies, Spearman correlation matrices were constructed separately for untreated and methotrexate-treated patients. Correlation heatmaps were generated to visualize the structure and strength of pairwise associations.

To further examine conditional dependencies and network organization, regularized partial correlation networks were estimated using the Extended Bayesian Information Criterion graphical least absolute shrinkage and selection operator (EBICglasso) method with a tuning parameter (γ) of 0.5. Networks were constructed independently for each treatment group. Edge weights represent regularized partial correlations, and only non-zero edges were retained in the final visualization. Network density was quantified as the proportion of non-zero edges relative to the total possible edges.

Edge accuracy and centrality stability were evaluated using nonparametric bootstrap procedures, including case-dropping methods (1000 resamples). Edge-weight accuracy was assessed based on bootstrap-derived confidence intervals, while centrality stability was examined using case-dropping bootstrap methods.

A formal comparison between networks was performed using the Network Comparison Test (NCT) based on permutation testing (1000 permutations), assessing differences in global strength and overall network structure between groups.

All statistical analyses and network estimations were performed using JASP software (version 0.19.3) and R (version 4.5.0), including the bootnet (version 1.8) and NetworkComparisonTest (version 2.2.3) packages.

Given the retrospective design, predefined inclusion and exclusion criteria were applied to minimize selection bias, and consecutive eligible patients were included to reduce the risk of arbitrary sampling. Stratification according to methotrexate exposure was based on treatment status at the time of hospitalization. Adjustment for all potential confounders was not feasible due to sample size constraints; however, baseline comparability between groups was formally evaluated. Network analyses were conducted separately within each treatment group. Given the relatively small size of the MTX-treated subgroup, network estimates were interpreted with caution, particularly for centrality measures known to be unstable in smaller samples; accordingly, betweenness and closeness were not interpreted.

### 4.4. Ethical Considerations

The study was conducted in accordance with the Declaration of Helsinki and approved by Ethics Committee of Bihor County Emergency Clinical Hospital, Oradea, Romania (Approval No. 31133/11 October 2024).

## 5. Conclusions

In this investigation, MTX-treated patients showed fewer cross-domain correlations and retained cross-domain network edges between inflammatory and metabolic biomarkers, while several intra-domain relationships remained evident. However, formal network comparison did not identify statistically significant differences in network structure or global strength between groups.

Given the relatively small size of the MTX-treated subgroup, which may limit statistical power and affect the stability of correlation estimates and network models, these findings should be interpreted with caution and considered exploratory and hypothesis-generating rather than indicative of treatment effects.

Network-based approaches may provide complementary insight into systemic processes in psoriasis; however, causal inferences cannot be drawn. Further prospective and adequately powered studies are required to clarify the temporal dynamics, reproducibility, and clinical relevance of inflammatory–metabolic interactions during systemic therapy, and to determine whether these patterns translate into meaningful clinical outcomes.

## Figures and Tables

**Table 1:** Baseline characteristics according to methotrexate exposure (N = 132).

Variable	No Therapy (n = 101)	MTX (n = 31)	*p*-Value
Age (years), median (IQR)	59 (50–68)	59 (50–68)	0.386
Male sex, n (%)	62 (61.4%)	17 (54.8%)	0.515
Urban residence, n (%)	55 (54.5%)	13 (41.9%)	0.222
Hypertension, n (%)	56 (55.4%)	23 (74.2%)	0.063
Obesity, n (%)	49 (48.5%)	15 (45.2%)	0.774
Diabetes mellitus, n (%)	30 (29.7%)	9 (29.0%)	0.943
Dyslipidemia, n (%)	37 (36.6%)	10 (32.3%)	0.656
Other cardiovascular disease, n (%)	36 (35.6%)	9 (29.0%)	0.497
Chronic kidney disease, n (%)	14 (13.9%)	3 (9.7%)	0.543
Metabolic syndrome, n (%)	42 (41.6%)	11 (35.5%)	0.544
Disease onset (years), median (IQR)	10 (5–15)	12 (5.5–21.5)	0.252
Length of hospital stay (days), median (IQR)	5 (3–7)	5 (3.5–7)	0.581
Number of hospitalizations, median (IQR)	1 (1–1)	1 (1–1)	0.006

Continuous variables are presented as median (interquartile range) and compared using the Mann–Whitney U test; categorical variables are presented as counts (percentages) and compared using the χ^2^ test. MTX, methotrexate.

**Table 2:** Inflammatory and metabolic biomarkers according to methotrexate exposure.

Variable	No Therapy (n = 101)	MTX (n = 31)	*p*-Value
CRP (mg/L)	6.8 (3.4–15.25)	6.2 (3.73–13.45)	0.830
ESR (mm/h)	18 (12–33)	21 (15–45.5)	0.234
NLR	3.17 (1.99–5.28)	2.42 (1.91–3.04)	0.035
SII	700.4 (437.5–1306.5)	612.4 (531.3–871.5)	0.569
Leukocytes (10^3^/µL)	8.87 (7.04–11.68)	8.66 (6.37–13.85)	0.813
Neutrophils (10^3^/µL)	5.9 (4.3–8.36)	4.94 (3.94–6.91)	0.169
Lymphocytes (10^3^/µL)	1.83 (1.22–2.42)	2.01 (1.56–2.73)	0.200
Platelets (10^3^/µL)	248.5 (183–286)	262 (221–340.1)	0.072
Glucose (mg/dL)	111 (94–131)	95 (84–108.5)	0.004
HDL (mg/dL)	38 (29–47)	42 (34–47)	0.172
TyG	8.77 (8.40–9.19)	8.79 (8.34–8.96)	0.629
METS-IR	39.33 (32.39–50.34)	38.68 (33.50–45.27)	0.691
AIP	0.51 (0.26–0.68)	0.47 (0.32–0.69)	0.947

Data are presented as median (interquartile range). Group comparisons were performed using the Mann–Whitney U test. MTX, methotrexate; CRP, C-reactive protein; ESR, erythrocyte sedimentation rate; NLR, neutrophil-to-lymphocyte ratio; SII, systemic immune-inflammation index; HDL, high-density lipoprotein; TyG, triglyceride–glucose index; METS-IR, metabolic score for insulin resistance; AIP, atherogenic index of plasma.

**Table 3:** Inflammatory and cross-domain spearman correlations according to methotrexate exposure.

Marker Pair	No Therapy (n = 101) ρ	*p*-Value	MTX (n = 31) ρ	*p*-Value
NLR–SII	0.796	<0.001	0.852	<0.001
CRP–ESR	0.740		0.816	
CRP–TyG	0.309	0.002	0.308	0.098
CRP–AIP	0.255	0.011	0.337	0.069
ESR–TyG	0.271	0.006	0.245	0.184
ESR–METS-IR	0.201	0.044	0.377	0.037
ESR–AIP	0.165	0.099	0.357	0.049
SII–TyG	0.212	0.034	0.145	0.434

CRP, C-reactive protein; ESR, erythrocyte sedimentation rate; TyG, triglyceride–glucose index; METS-IR, metabolic score for insulin resistance; SII, systemic immune-inflammation index; NLR, neutrophil-to-lymphocyte ratio; AIP, atherogenic index of plasma.

**Table 4:** Global network characteristics according to methotrexate exposure.

Network Parameter	No Therapy (n = 101)	MTX (n = 31)
Number of nodes	6	6
Non-zero edges	12/15	9/15
Network sparsity	0.20	0.40
Sum of absolute edge weights	2.47	2.59
Mean edge weight	0.206	0.288

Edge weights represent EBICglasso regularized partial correlations.

## Data Availability

The original contributions presented in this study are included in the article. Further inquiries can be directed to the corresponding authors.

## Abbreviations

The following abbreviations are used in this manuscript:

AIP	Atherogenic index of plasma
CBC	Complete blood count
CRP	C-reactive protein
EBICglasso	Extended Bayesian information criterion graphical least absolute shrinkage and selection operator (regularized partial correlation network method)
ESR	Erythrocyte sedimentation rate
HDL	High-density lipoprotein cholesterol
IL-6	Interleukin-6
IL-17	Interleukin-17
IL-22	Interleukin-22
IL-23	Interleukin-23
JASP	JASP statistical software
METS-IR	Metabolic score for insulin resistance
MTX	Methotrexate
NLR	Neutrophil-to-lymphocyte ratio
SD	Standard deviation
SII	Systemic immune-inflammation index
Th17	T helper 17 cells
TNF-α	Tumor necrosis factor-alpha
TyG	Triglyceride–glucose index
**γ**	Tuning parameter (gamma) used in EBICglasso regularization
